# Surveillance MRI is associated with improved survival in patients with primary sclerosing cholangitis

**DOI:** 10.1097/HC9.0000000000000442

**Published:** 2024-05-02

**Authors:** Natassia Tan, Natalie Ngu, Thomas Worland, Tanya Lee, Tobie Abrahams, Elliot Freeman, Nicholas Hannah, Kathryn Gazelakis, Richie G Madden, Kate D Lynch, Zina Valaydon, Siddharth Sood, Anouk Dev, Sally Bell, Alexander J Thompson, John Nik Ding, Amanda J Nicoll, Ken Liu, Keval Pandya, Paul Gow, John Lubel, William Kemp, Stuart K Roberts, Ammar Majeed

**Affiliations:** 1 Department of Gastroenterology and Hepatology, The Alfred, Melbourne, Australia; 2 Central Clinical School, Monash University, Melbourne, Australia; 3 Department of Gastroenterology and Hepatology, Monash Health, Melbourne, Australia; 4 Department of Gastroenterology and Hepatology, Austin Health, Melbourne, Australia; 5 Department of Gastroenterology and Hepatology, St Vincent’s Health, Melbourne, Australia; 6 Department of Gastroenterology and Hepatology, Melbourne Health, Melbourne, Australia; 7 University of Melbourne, Melbourne, Australia; 8 Department of Gastroenterology and Hepatology, Western Health, Melbourne, Australia; 9 Department of Gastroenterology and Hepatology, Royal Adelaide Hospital, Adelaide, Australia; 10 Department of Gastroenterology and Hepatology, Eastern Health, Melbourne, Australia; 11 AW Morrow Gastroenterology and Liver Centre, Royal Prince Alfred Hospital, Sydney, Australia

## Abstract

**Background:**

The benefits of regular surveillance imaging for cholangiocarcinoma in patients with primary sclerosing cholangitis (PSC) are unclear. Hence, we aimed to evaluate the impact of regular magnetic resonance cholangiopancreatography (MRCP) on outcomes of patients with PSC in Australia, where the practice of MRCP surveillance is variable.

**Methods:**

The relationship between MRCP surveillance and survival outcomes was assessed in a multicenter, retrospective cohort of patients with PSC from 9 tertiary liver centers in Australia. An inverse probability of treatment weighting approach was used to balance groups across potentially confounding covariates.

**Results:**

A total of 298 patients with PSC with 2117 person-years of follow-up were included. Two hundred and twenty patients (73.8%) had undergone MRCP surveillance. Regular surveillance was associated with a 71% reduced risk of death on multivariate weighted Cox analysis (HR: 0.29, 95% CI: 0.14–0.59, *p* < 0.001) and increased likelihood of having earlier endoscopic retrograde cholangiopancreatography from the date of PSC diagnosis in patients with a dominant stricture (*p* < 0.001). However, survival posthepatobiliary cancer diagnosis was not significantly different between both groups (*p* = 0.74). Patients who had surveillance of less than 1 scan a year (n = 41) had comparable survival (HR: 0.46, 95% CI 0.16–1.35, *p* = 0.16) compared to patients who had surveillance at least yearly (n = 172).

**Conclusions:**

In this multicenter cohort study that employed inverse probability of treatment weighting to minimize selection bias, regular MRCP was associated with improved overall survival in patients with PSC; however, there was no difference in survival after hepatobiliary cancer diagnosis. Further prospective studies are needed to confirm the benefits of regular MRCP and optimal imaging interval in patients with PSC.

## Graphical abstract

**Figure:**
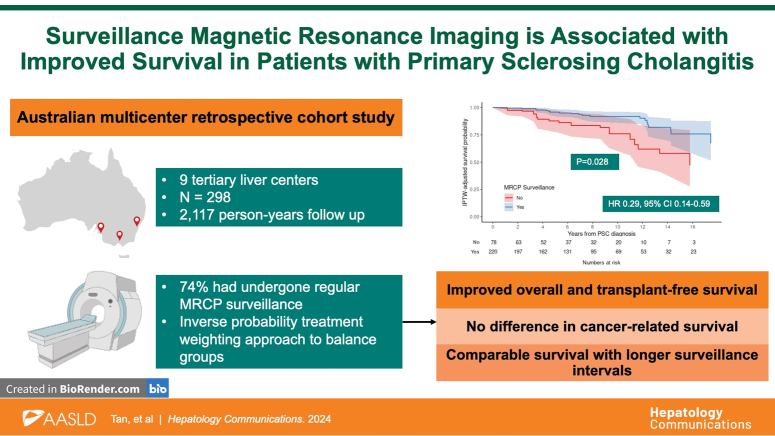

## INTRODUCTION

Primary sclerosing cholangitis (PSC) is an orphan, chronic inflammatory disease with a predilection for middle-aged men. It classically manifests as progressive multifocal bile duct stricturing, with a risk for the development of end-stage biliary cirrhosis.^1,2^ There is a strong association with inflammatory bowel disease (IBD) and malignancies of the colon and hepatobiliary system.^3,4^ Among these cancers, cholangiocarcinoma (CCA) is the most common and carries a poor prognosis with a 5-year survival of less than 20%.^5^ Only a small proportion of patients with early-stage diagnosis may be considered for curative treatment with resection or liver transplant (LT).^3^

Due to the increased risk of colorectal cancer in patients with PSC and concomitant IBD, annual colonoscopies are well recognized as a surveillance strategy in multiple international guidelines.^6–8^ However, evidence-based strategies for the surveillance of CCA are lacking and it is unclear if imaging surveillance does improve cancer-related survival based on the current literature available. A recent Swedish prospective cohort study of an unselected group of 512 patients with PSC showed that annual imaging and tumor marker surveillance was not associated with improved cancer-related survival, although the authors stated that it was not possible to determine the true impact of this.^9^ In contrast, international retrospective cohort studies have demonstrated that regularly scheduled imaging improved OS and cancer-related outcomes.^10,11^ A population registry-based study on 2588 patients with PSC and IBD also found that annual imaging was associated with a twofold reduction in the risk of hepatobiliary cancer-related death; however, this benefit was not sustained after the exclusion of CCA cases diagnosed within the first year of PSC diagnosis.^12^

International guidelines have recently been updated to recommend annual surveillance imaging for CCA^13–15^ while acknowledging the paucity of data to support this recommendation. Magnetic resonance cholangiopancreatography (MRCP) is preferred to ultrasound in view of its superior accuracy,^14,16^ although European guidelines recommend annual imaging with either modality. In view of the limited literature and variation in guideline recommendations, surveillance strategies currently vary significantly across different centers.^10^ Hence, we aimed to evaluate the impact of regular MRCP on the clinical outcomes of patients with PSC in Australia, where the practice of surveillance is variable.

## METHODS

We evaluated MRCP surveillance, clinical, and outcome data using a multicenter, retrospective cohort of patients with PSC diagnosed between January 1, 2000 and March 30, 2021 from 9 tertiary liver centers in Australia as described.^17^ This study was approved by the Alfred Hospital Human Research Ethics Committee in Melbourne (Project 353/19), Australia. “Surveillance” is defined as the monitoring of the occurrence of a disease in a population that is at risk.^11^

Individual centers manually reviewed the medical records to assess the indication of each MRCP performed. Patients were categorized into the MRCP surveillance group if they ever had periodic MRCP imaging over the follow-up period for the purposes of cancer surveillance, even if they subsequently had imaging at a certain time point to investigate clinical change. Patients were categorized into the no surveillance group if they never had MRCP imaging for the purposes of cancer surveillance and only had imaging in response to clinical deterioration or concern. All surveillance MRCPs did not include i.v. contrast as a part of the study. Information on the frequency of MRCP surveillance was divided categorically into groups who had MRCP surveillance at least yearly and those who had MRCP surveillance at intervals longer than a year apart.

A dominant stricture (DS) was defined as a stenosis of the common bile duct or common hepatic duct with a diameter of ≤1.5 mm and/or ≤1.0 mm in a hepatic duct (within 2 cm of the bifurcation).^18^ This definition was used as data collection had preceded the new working definition published in 2021.^19^ Clinical data on endoscopic retrograde cholangiopancreatography (ERCP) was only available for patients with a diagnosis of DS. Clinical data regarding diagnosis, staging, and treatment were collected in patients diagnosed with hepatobiliary cancers (CCA and/or gallbladder adenocarcinoma). Cancer staging was based on the American Joint Committee on Cancer staging system.

Patients from the original cohort^17^ were excluded if there was insufficient data collected. The Model of End-Stage Liver Disease (MELD) score at the last follow-up was calculated with the following calculation: 0.957 × ln(creatinine) + 0.378 × ln(bilirubin) + 1.120 × ln(INR) + 0.643. Incomplete data from the individual variables were imputed through multivariate imputation by chained equations with the mice package version 3.15.0 on R version 4.2.2.^20^ The primary end point was overall survival from the date of PSC diagnosis, and secondary end points included transplant-free survival and cancer-related survival. We also evaluated the impact of the frequency of surveillance imaging on the above end points. A sub-analysis was performed after excluding all patients with small duct PSC, and these results are presented in the supplementary document provided.

### Statistical analysis

Nonparametric continuous variables were presented as medians and IQR and analyzed with the Mann-Whitney *U* test for skewed distributions. Categorical data were presented as percentages and compared using chi-square or Fisher exact test. An inverse probability of treatment weighting (IPTW) approach was used to balance potentially confounding covariates between both groups. This was done to achieve a standardized mean difference of less than 0.10 for all confounding covariates.

An IPTW-weighted Cox proportional hazards model was conducted to assess the risk of death. A competing risk analysis (Fine-Gray model) was conducted whereby the failure event was represented by the death of any cause, whereas LT was considered the competing event. The Kaplan-Meier method with log-rank test was used to estimate and detect differences in survival time from diagnosis of PSC and CCA in the weighted cohorts. Estimated survival probabilities were presented as median and IQR, or mean and SD if a 50% survival probability was not reached. A two-tailed *p*-value of <0.05 was considered statistically significant, and 95% CIs are reported where appropriate. Data were analyzed using R (version 4.2.2).

## RESULTS

### Unweighted study population

Two hundred and ninety-eight patients with PSC were included from the original cohort after the exclusion of patients with missing data. Twenty-nine patients (9.7%) had a small duct phenotype at diagnosis. Patients were followed up for a median of 6 (IQR: 3–11) years, resulting in a total of 2117 person-years of follow-up. During the follow-up period, 46 (15.4%) had a LT and 35 (11.7%) died. Seventy-seven patients (25.8%) were diagnosed with a DS. Thirteen patients (7.6%) were diagnosed with hepatobiliary cancers, with a median of 22 (IQR: 4–38) weeks of follow-up from cancer diagnosis.

Two hundred and twenty patients (73.8%) of the cohort had undergone regular MRCP surveillance. Patients who underwent regular surveillance were younger at PSC diagnosis (35.5 vs. 45.5 y, *p* = 0.003), more likely to be found to have a DS (30.0% vs. 14.1%, *p* = 0.009), concomitant IBD (75.5% vs. 57.7%, *p* = 0.005), have a higher MELD score (median, 8.5 vs. 7.5, *p* = 0.01), serum bilirubin (median, 15 vs. 11 μmol/L, *p* = 0.003) and serum alkaline phosphatase (median, 166.5 vs. 111 IU/L, *p* = 0.002). The frequency of endoscopic intervention was similar in patients with DS in both groups (19% vs. 10.2%, *p* = 0.30). The frequency of symptomatic DS was also similar in both groups (76% vs. 75%, *p* = 0.93). Table 1 demonstrates the demographics and clinical characteristics of patients who had regular surveillance and those who did not.

**TABLE 1:** Demographics and clinical characteristics of unweighted cohorts of patients with PSC stratified by MRCP surveillance status

	MRCP surveillance (N = 220)	No MRCP surveillance (N = 78)	*p*	Standardized mean difference
Age at PSC diagnosis, y (median, IQR)	35.5 (22–51.3)	45.5 (30–54.8)	**0.003**	**0.395**
Male, N (%)	147 (66.8)	43 (55.1)	0.088	**0.241**
Large duct, N (%)	201 (91.3)	68 (87.2)	0.403	**0.134**
Inflammatory bowel disease, N (%)	166 (75.5)	45 (57.7)	**0.005**	**0.383**
Dominant stricture, N (%)	66 (30)	11 (14.1)	**0.009**	**0.391**
ERCP, N (%)	42 (19)	8 (10.2)	0.634	**0.305**
Cirrhosis, N (%)	84 (38.2)	31 (39.7)	0.914	0.032
Death, N (%)	20 (9.1)	15 (19.2)	**0.029**	**0.294**
LT, N (%)	33 (15)	13 (16.7)	0.867	0.046
HBCa, N (%)	11 (5)	2 (2.6)	0.560	0.128
CCA	9 (81.8)	2 (100)	—	—
GB	2 (9.2)	0	—	—
Serum ALP^a^, IU/L (median, IQR)	166.5 (100–293)	111 (79–215)	**0.002**	**0.285**
Serum bilirubin^a^, μmol/L (median, IQR)	15 (9–25.5)	11 (7–19)	**0.003**	**0.285**
MELD score^a^ (median, IQR)	8.47 (7.34–10.2)	7.50 (6.43–9.76)	**0.014**	**0.178**

Bold values are statistically significance.

a Values taken at last follow-up.

Abbreviations: ALP, alkaline phosphatase; CCA, cholangiocarcinoma; ERCP, endoscopic retrograde cholangiopancreatography; GB, gallbladder adenocarcinoma; HBCa, hepatobiliary cancer; LT, liver transplant; MELD, Model of End-Stage Liver Disease; MRCP, magnetic resonance cholangiopancreatography; PSC, primary sclerosing cholangitis.

### Results: influence of regular MRCP surveillance

#### Weighted study population

IPTW was performed with age of diagnosis, phenotype at diagnosis, gender, MELD score, concomitant IBD, presence of cirrhosis or DS, LT, and serum alkaline phosphatase as demonstrated in Supplemental Figure S1, http://links.lww.com/HC9/A883. The demographics and clinical characteristics of the IPTW cohort are demonstrated in Table 2. Even though weighting was not performed for ERCP, the frequency of this amongst patients with DS were not statistically significant (16.2% vs. 18.3%, *p* = 0.66).

**TABLE 2:** Demographics and clinical characteristics of inverse probability treatment weighted cohorts of patients with PSC stratified by MRCP surveillance status

	MRCP surveillance	No MRCP surveillance	*p*	Standardized mean difference
Age of PSC diagnosis, y (median, IQR)	40 (23–53)	36 (28–52)	0.798	0.022
Male, %	63.5	61.2	0.740	0.050
Dominant stricture, %	25.9	27.9	0.790	0.046
Type, %	91.3	89.2	0.576	0.072
IBD, %	70.3	68.8	0.816	0.033
Cirrhosis, N (%)	38.8	38.1	0.922	0.014
LT, N (%)	15.5	17.6	0.720	0.054
Serum ALP (median, IQR)	154.8 (93.9–268)	122 (87.8–261)	0.290	0.027
MELD score (median, IQR)	8.41 (7.22–10)	7.5 (6.43–10.5)	0.254	0.037
ERCP^a^ (%)	16.2	18.3	0.663	**0.176**

Bold values are statistically significance.

a Not weighted.

Abbreviations: ALP, alkaline phosphatase; ERCP, endoscopic retrograde cholangiopancreatography; LT, liver transplant; MELD, Model of End-Stage Liver Disease score; MRCP, magnetic resonance cholangiopancreatography; PSC, primary sclerosing cholangitis.

#### Overall survival

**FIGURE 1:**
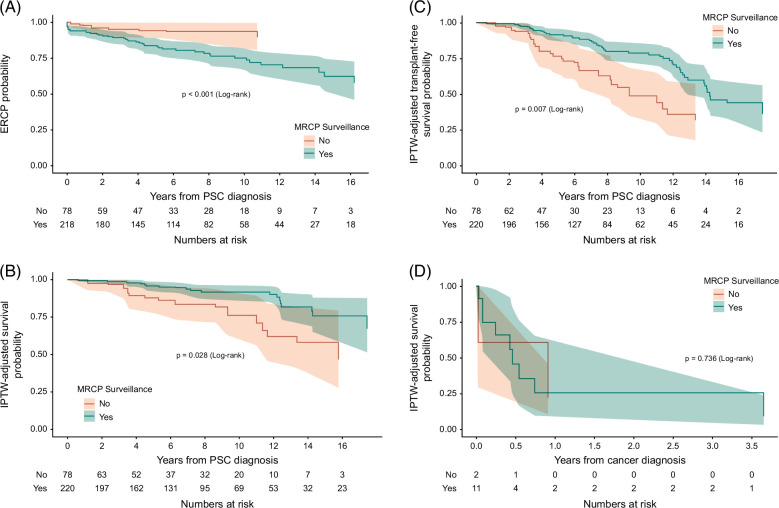
(A) Weighted Kaplan-Meier curve for time to ERCP from PSC diagnosis*, (B) Weighted Kaplan-Meier curve for overall survival, (C) Weighted Kaplan-Meier curve for transplant-free survival, (D) Weighted Kaplan-Meier curve for survival post-cancer diagnosis. *Two ERCP dates are missing from the surveillance group. Abbreviations: ERCP indicate endoscopic retrograde cholangiopancreatography, IPTW; inverse probability treatment weighting, MRCP; magnetic resonance imaging with cholangiopancreatography, PSC; primary sclerosing cholangitis.

During follow-up, 20 (9.1%) patients in the surveillance group and 15 (19.2%) in the nonsurveillance group died. Four (20%) patients in the surveillance group and 5 (33%) patients in the nonsurveillance group died from end-stage liver disease. Seven (35%) patients in the surveillance group and 2 (13%) patients in the nonsurveillance group died from hepatobiliary cancer. Only 1 (5%) patient in the surveillance group died from HCC. There were no deaths from colorectal cancers in both groups. There was no statistically significant difference in the cause of death between both groups, as described in Supplemental Table 1, http://links.lww.com/HC9/A883 (*p* = 0.08). Patients in the surveillance group with a DS were more likely to have had an ERCP at an earlier time point from diagnosis of PSC compared to patients in the nonsurveillance group on Kaplan-Meier analysis of the weighted cohorts (*p* < 0.001, Figure 1A). There was no significant difference between indications of ERCP in both groups, as described in Supplemental Table 2, http://links.lww.com/HC9/A883 (*p* = 0.20), although the proportion of patients in the surveillance group who had an ERCP for investigation of malignancy (26.5% vs. 14.3%) or interrogation of an asymptomatic DS was numerically higher (17.6% vs. 0%).

Improved overall survival from the time of PSC diagnosis was demonstrated for the surveillance group on Kaplan-Meier analysis of the weighted cohorts (*p* = 0.03, Figure 1B). The mean survival of patients in the surveillance group was 18.3 (SD ± 0.47) compared with 15.1 (SD ± 0.61) years in the group without regular surveillance. On Cox proportional hazards model univariate analysis in the weighted cohorts, regular MRCP surveillance was significantly associated with a 60% (HR: 0.40, 95% CI: 0.19–0.81, *p* = 0.01) reduction in risk of death. MRCP surveillance remained significant on multivariate analysis (HR: 0.29, 95% CI: 0.14–0.59, *p* < 0.001), as shown in Supplemental Table 3, http://links.lww.com/HC9/A883.

**FIGURE 2:**
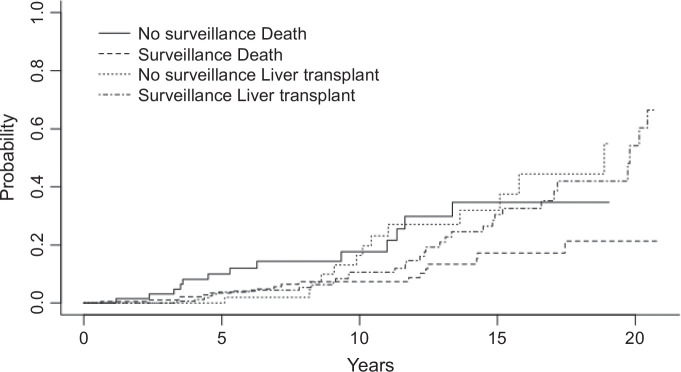
Cumulative incidence function curve using Fine-Gray competing risk analysis.

In a competing risk survival analysis, with LT as a competing risk for death, the cumulative incidence of death remained significantly lower in the surveillance group (HR: 0.33, 95% CI: 0.17–0.64, *p* = 0.002, Supplemental Table 4, http://links.lww.com/HC9/A883 and Figure 2).

#### Transplant-free survival

A total of 33 (15%) patients in the surveillance group and 13 (16.7%) in the nonsurveillance group received LT during the study period. Transplant-free survival was significantly improved in patients who had regular MRCP surveillance (*p* = 0.01, Figure 1C). Regular MRCP surveillance remained a significant factor in multivariate Cox regression with a 49% reduction in risk of LT or death (HR: 0.51, 95% CI: 0.3–0.86, *p* = 0.012).

#### Hepatobiliary cancer-related survival and outcomes

The event rate of development of hepatobiliary cancer was 6.1 (95% CI: 3.2–10.5) per 1000 person-years at risk in the cohort. In patients who were diagnosed with hepatobiliary cancer, 11 (5%) were in the surveillance group and 2 (2.6%) were in the nonsurveillance group (*p* = 0.56). Only 2 (15.3%) patients were asymptomatic at the time of diagnosis, and 8 (61.5%) patients had stage IV disease at the time of diagnosis. Only 1 patient in the surveillance group diagnosed with gallbladder adenocarcinoma was able to receive treatment with curative intent, while all other patients received palliative chemotherapy or best supportive care. There was no significant difference in survival post-cancer diagnosis with a median of 5.5 (IQR: 0.95–43.7) months in the surveillance group compared to 10.9 (IQR: 0.2–10.9) months in the nonsurveillance group (*p* = 0.74) as demonstrated in Figure 1D on weighted Kaplan-Meier analysis. Table 3. illustrates the clinical characteristics, cancer treatment, and clinical outcomes of patients diagnosed with hepatobiliary cancer during the study period.

**TABLE 3:** Clinical characteristics of patients diagnosed with hepatobiliary cancer

	MRCP surveillance	Cancer	Symptoms	Stage	Treatment	Death	Time from diagnosis to death (wk)
1	Yes	IHC	Yes	IV	BSC	Yes	4
2	Yes	EHC	Yes	NA^a^	BSC	Yes	1.5
3	Yes	IHC	No	IV	Palliative chemotherapy	No	—
4	Yes	GBC	No	II	Curative surgery and adjuvant chemotherapy	No	—
5	Yes	EHC	Yes	IV	Palliative chemotherapy	Yes	28
6	Yes	IHC	Yes	IV	NA^b^	Yes	4
7	Yes	IHC	Yes	IV	Palliative chemotherapy	Yes	38
8	Yes	GBC	Yes	IV	Palliative chemotherapy and radiotherapy	Yes	190
9	Yes	IHC	Yes	IV	Palliative chemotherapy	Yes	22
10	Yes	EHC	Yes	IIIA	BSC	Yes	12
11	Yes	EHC	Yes	IIIB	BSC	Yes	23
12	No	IHC	NA	IV	Palliative chemotherapy	Yes	1
13	No	IHC	Yes	II	BSC	Yes	47

a Patient not staged as developed acute liver failure secondary to portal vein thrombosis and was palliated.

b Unknown as patient was treated in another hospital.

Abbreviations: BSC, best supportive care; IHC, intrahepatic cholangiocarcinoma; EHC, extrahepatic cholangiocarcinoma; GBC, gallbladder adenocarcinoma; MRCP, magnetic resonance cholangiopancreatography; NA, unknown.

### Outcomes: influence of MRCP surveillance interval

#### Unweighted study population

The MRCP surveillance cohort was stratified according to interval time between MRCP surveillance into either annual MRCP (n = 172) or those having less than 1 MRCP a year (n = 41). There were no clinically significant differences detected between both cohorts as demonstrated in Supplemental Table 5, http://links.lww.com/HC9/A883.

### Overall survival according to MRCP interval

#### Weighted study population

IPTW was performed with the age of diagnosis, phenotype at diagnosis, gender, MELD score at the time of last follow-up, concomitant IBD, presence of DS, LT, and serum alkaline phosphatase as demonstrated in Supplemental Figure S2, http://links.lww.com/HC9/A883. The demographics and clinical characteristics of the IPTW cohort are demonstrated in Supplemental Table 6, http://links.lww.com/HC9/A883, with no significant difference between the groups identified.

**FIGURE 3:**
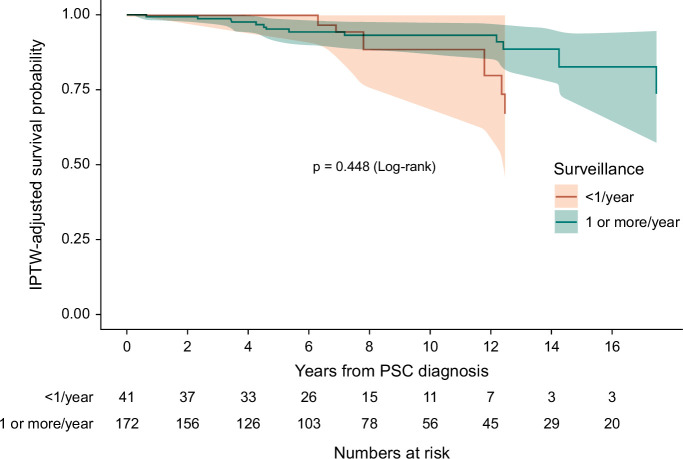
Kaplan-Meier curve for survival stratified by surveillance intervals. Abbreviations: IPTW; inverse probability treatment weighting, MRCP; magnetic resonance imaging with cholangiopancreatography, PSC; primary sclerosing cholangitis.

On weighted Cox proportional hazards model univariate analysis, annual MRCP surveillance was not associated with improved survival compared to patients who had surveillance imaging less frequently (HR: 0.46, 95% CI: 0.16–1.35, *p* = 0.16). There was no significant difference in survival time from PSC diagnosis between the 2 groups on weighted Kaplan-Meier analysis (*p*=0.45) as shown in Figure 3. The mean survival of patients in the annual MRCP surveillance group was 18.3 (SD ± 0.54) years compared with 15.7 (SD ± 0.62) years in the group with less frequent surveillance.

## DISCUSSION

In this multicenter, retrospective study that employed IPTW to minimize selection bias, we found that a survival benefit was derived from regular MRCP surveillance in a well-described Australian cohort of patients with PSC. However, this does not seem to be related to improved outcomes from early cancer diagnosis and management. In addition, we demonstrated that imaging performed at intervals even greater than yearly does not compromise this survival benefit.

Our study draws similarities to previous retrospective studies where natural “surveillance groups” are formed due to a variety of factors such as patient and physician choice, clinical characteristics of disease, and surveillance practice at the centers involved.^10,11^ A previous single tertiary center retrospective cohort study demonstrated significant improvement in survival and cancer-related outcomes postdiagnosis with imaging surveillance, although patient health literacy and insurance status may have played a part as patients were given a choice to participate.^11^ Another reason for being able to detect improved cancer-related outcomes may have been due to the large proportion of patients in the cohort diagnosed with hepatobiliary cancer (9.5%), with the majority (40%) of patients being asymptomatic at diagnosis.^11^ In this setting, patients were more likely to be diagnosed at an earlier stage and amenable to curative. However, 93% of patients diagnosed with hepatobiliary cancer in our study were symptomatic, which suggests that in our cohort, imaging surveillance itself was not sufficient to detect early asymptomatic cancers. An important aspect to highlight was that all surveillance MRCPs in our study did not include i.v. contrast, which is likely not as accurate at detecting early cancers.

Even with our smaller cohort, our findings mirror those of a recent large, international multicentre study by Bergquist et al with 2675 patients demonstrating improved survival in patients who underwent regular imaging and/or ERCP.^10^ Only a small proportion of their cohort developed CCA (4.2%), which is in parallel with ours (3.7%). Bergquist et al reported 69% of patients who developed CCA had symptomatic disease at the time of diagnosis, which would have led to imaging and further interrogation with ERCP in most cases. This coincided with our cohort, where 84.7% were symptomatic and 61.5% had stage IV disease at the time of diagnosis. Both our findings demonstrate retention in survival benefit postadjustment for CCA, highlighting again that this survival benefit may not be entirely attributable to early diagnosis.^10^

Although the proportion of patients who received ERCP was not significantly different between both groups, we demonstrated that patients who had regular surveillance were likely to undergo ERCP at an earlier time point from PSC diagnosis. There was also an increased proportion of ERCP performed in the surveillance group for the investigation of potential malignancy and interrogation of an asymptomatic DS. This data may allow us to speculate that some patients receiving ERCP in the surveillance group may be triggered by progressive, high-grade strictures on serial imaging with associated higher markers of cholestasis rather than a “reactive” clinical approach to worsening symptoms or liver function tests. This potentially led to earlier endoscopic treatment with preservation of liver parenchyma, which has the potential for improving survival as supported by older studies.^21–23^

We found that there was no significant difference in cause of death between both groups in our study, with the majority dying from end-stage liver disease or hepatobiliary cancer. However, the overall number of deaths was small. This suggests that early endoscopic treatment and regular MRCP imaging, which could also be a surrogate marker for increased health care engagement and improved adherence, may prolong survival but ultimately not change the natural history of PSC.

We acknowledge that the reason for survival benefit derived in the surveillance cohort is not entirely clear despite our assumptions. However, the survival benefit demonstrated is robust and is supported by recent literature in this area. We may have also failed to produce a cancer-related survival benefit due to type II error that has been shown in larger population cohorts.^12^ Nevertheless, our results are also similar to the only prospective cohort study that was recently published by Villard et al, again demonstrating that regular annual MRCP and serum carbohydrate antigen 19-9 for CCA surveillance over a 5-year period did not have improved cancer-related outcomes.^9^ Although this was a relatively large cohort (N = 512), only 2% developed CCA during the follow-up period. Furthermore, Villard et al showed that severe and/or progressive bile duct changes on MRCP and increased levels of serum CA 19-9 were significantly associated with a higher risk of development of high-grade dysplasia or CCA. This provides important information that this subgroup of patients may warrant more frequent imaging and that monitoring for progressive changes plays a part in potentially delaying the clinical progression of the disease.

It is also important to know that patients diagnosed with small duct PSC are at risk of progression to large duct PSC over time, which has been demonstrated in other studies.^24^ There is not enough data currently to determine optimal imaging intervals for patients with small duct PSC to evaluate for progression, but current European guidelines recommend imaging every 3 years in patients with stable liver function tests.^13^

This is the first large-scale study from the Southern Hemisphere investigating MRCP surveillance practices and the influence of this on the outcomes of patients with PSC. To minimize selection bias in this observational study, we employed IPTW and a competing risk analysis to demonstrate that regular MRCP surveillance itself does influence the clinical course of PSC in a positive way. We acknowledge the limitations of our study being a retrospective analysis, with real-world challenges of missing data points, which we have attempted to rectify with multivariate imputation models. Due to a small patient cohort and low incidence of hepatobiliary cancers over the relatively short follow-up period, our study had inadequate power to detect an improvement in cancer-related survival. As surveillance practices are variable in Australia, serum carbohydrate Ag 19-9 levels were not routinely collected and hence could not be included in the analysis. We also cannot completely account for misclassification bias, where patients may have mistakenly been classified into the surveillance group when they were having imaging in response to clinical change. However, this was less likely as the surveillance practices were considered over their whole follow-up period, and individual case records were scrutinized.

As with all studies looking at the impact of surveillance on cancer survival, lag-time bias, lead-time bias and immortal-time bias are impossible to eliminate. However, our study did not identify cancer-related survival benefits, which reduces the effect of those potential confounders on our results. We also were unable to do a cost-effectiveness analysis, as we acknowledge frequent MRCP imaging is expensive and results in lost work productivity for patients. However, annual imaging may not necessarily be warranted as our study suggests less frequent imaging may still provide a survival benefit, which may allow for a more cost-effective strategy with longer intervals between surveillance imaging. Future prospective studies are needed to interrogate this issue as we acknowledge that the number of patients who received surveillance imaging at longer intervals than annually were relatively small with a low event rate.

## CONCLUSIONS

Our study showed that regular MRCP surveillance in an Australian cohort of patients with PSC improved overall survival. This survival benefit was not from improving cancer-related outcomes but rather could have been due to improved health care engagement and detection of clinically relevant strictures that were amenable to earlier endoscopic intervention(s) to reduce cholestasis. Importantly, we also showed that annual imaging may not be critically important to provide a survival benefit and that surveillance intervals could be extended depending on the patient’s clinical circumstances. There is increasing evidence to show that surveillance MRCP does not translate to improved cancer-related survival. As such, there should be a shift in mindset towards patients with PSC having regular MRCP monitoring for disease progression rather than cancer surveillance, alongside the recommendations for hepatobiliary ultrasound for gallbladder adenocarcinoma and liver cancer surveillance.

## Supplementary Material

SUPPLEMENTARY MATERIAL

## References

[1] Hirschfield GM Karlsen TH Lindor KD Adams DH. Primary sclerosing cholangitis. Lancet. 2013;382:1587–1599. 23810223 10.1016/S0140-6736(13)60096-3

[2] Lazaridis KN LaRusso NF. Primary sclerosing cholangitis. N Engl J Med. 2016;375:1161–1170. 27653566 10.1056/NEJMra1506330 PMC5553912

[3] Rizvi S Eaton JE Gores GJ. Primary sclerosing cholangitis as a premalignant biliary tract disease: Surveillance and management. Clin Gastroenterol Hepatol. 2015;13:2152–2165. 26051390 10.1016/j.cgh.2015.05.035 PMC4618039

[4] Fung BM Lindor KD Tabibian JH. Cancer risk in primary sclerosing cholangitis: Epidemiology, prevention, and surveillance strategies. World J Gastroenterol. 2019;25:659–671. 30783370 10.3748/wjg.v25.i6.659 PMC6378537

[5] Grimsrud MM Folseraas T. Pathogenesis, diagnosis and treatment of premalignant and malignant stages of cholangiocarcinoma in primary sclerosing cholangitis. Liver Int. 2019;39:2230–2237. 31216595 10.1111/liv.14180

[6] Leong R Crispin C Koh C Wu B Kariyawasam V. Cancer Council Australia Surveillance Colonoscopy Guidelines Working Party. Clinical question: What is the most appropriate time interval for surveillance in IBD patients? In: Clinical practice guidelines for surveillance colonoscopy. Sydney: Cancer Council Australia; 2007.https://wiki.cancer.org.au/australia/Guidelines:Colorectal_cancer/Colonoscopy_surveillance

[7] American Society for Gastrointestinal Endoscopy Standards of Practice C Shergill AK Lightdale JR Bruining DH Acosta RD Chandrasekhara V. The role of endoscopy in inflammatory bowel disease. Gastrointest Endosc. 2015;81:1101–21 e1-13. 25800660 10.1016/j.gie.2014.10.030

[8] Annese V Daperno M Rutter MD Amiot A Bossuyt P East J. European evidence based consensus for endoscopy in inflammatory bowel disease. J Crohns Colitis. 2013;7:982–1018. 24184171 10.1016/j.crohns.2013.09.016

[9] Villard C Friis-Liby I Rorsman F Said K Warnqvist A Cornillet M. Prospective surveillance for cholangiocarcinoma in unselected individuals with primary sclerosing cholangitis. J Hepatol. 2023;78:604–613. 36410555 10.1016/j.jhep.2022.11.011

[10] Bergquist A Weismüller TJ Levy C Rupp C Joshi D Nayagam JS. Impact on follow-up strategies in patients with primary sclerosing cholangitis. Liver Int. 2022;43:127–138. 35535655 10.1111/liv.15286 PMC10084018

[11] Ali AH Tabibian JH Nasser-Ghodsi N Lennon RJ DeLeon T Borad MJ. Surveillance for hepatobiliary cancers in patients with primary sclerosing cholangitis. Hepatology. 2018;67:2338–2351. 29244227 10.1002/hep.29730

[12] Trivedi PJ Crothers H Mytton J Bosch S Iqbal T Ferguson J. Effects of primary sclerosing cholangitis on risks of cancer and death in people with inflammatory bowel disease, based on sex, race, and age. Gastroenterology. 2020;159:915–928. 32445859 10.1053/j.gastro.2020.05.049

[13] European Association for the Study of the Liver. Electronic address jee, European Association for the Study of the L. EASL Clinical Practice Guidelines on Sclerosing Cholangitis. J Hepatol. 2022;77:761–806. 35738507 10.1016/j.jhep.2022.05.011

[14] Bowlus CL Arrivé L Bergquist A Deneau M Forman L Ilyas SI. AASLD practice guidance on primary sclerosing cholangitis and cholangiocarcinoma. Hepatology. 2022;77:659–702. 36083140 10.1002/hep.32771

[15] Bowlus CL Lim JK Lindor KD. AGA clinical practice update on surveillance for hepatobiliary cancers in patients with primary sclerosing cholangitis: Expert review. Clin Gastroenterol Hepatol. 2019;17:2416–2422. 31306801 10.1016/j.cgh.2019.07.011

[16] Eaton JE Welle CL Bakhshi Z Sheedy SP Idilman IS Gores GJ. Early Cholangiocarcinoma detection with magnetic resonance imaging versus ultrasound in primary sclerosing cholangitis. Hepatology. 2021;73:1868–1881. 32974892 10.1002/hep.31575 PMC8177077

[17] Tan N Ngu N Worland T Lee T Abrahams T Pandya K. Epidemiology and outcomes of primary sclerosing cholangitis: an Australian multicentre retrospective cohort study. Hepatol Int. 2022;16:1094–1104. 35657479 10.1007/s12072-022-10356-1 PMC9525417

[18] Karlsen TH Folseraas T Thorburn D Vesterhus M. Primary sclerosing cholangitis - A comprehensive review. J Hepatol. 2017;67:1298–1323. 28802875 10.1016/j.jhep.2017.07.022

[19] Ponsioen CY Assis DN Boberg KM Bowlus CL Deneau M Thorburn D. Defining primary sclerosing cholangitis: Results from an international primary sclerosing cholangitis study group consensus process. Gastroenterology. 2021;161:1764–75 e5. 34384749 10.1053/j.gastro.2021.07.046

[20] van Buuren S Groothuis-Oudshoorn K. mice: Multivariate imputation by chained equations in R. J Stat Softw. 2011;45:1–67.

[21] Stiehl A Rudolph G Kloters-Plachky P Sauer P Walker S. Development of dominant bile duct stenoses in patients with primary sclerosing cholangitis treated with ursodeoxycholic acid: Outcome after endoscopic treatment. J Hepatol. 2002;36:151–156. 11830325 10.1016/s0168-8278(01)00251-3

[22] Stiehl A Rudolph G Sauer P Benz C Stremmel W Walker S. Efficacy of ursodeoxycholic acid treatment and endoscopic dilation of major duct stenoses in primary sclerosing cholangitis. An 8-year prospective study. J Hepatol. 1997;26:560–566. 9075663 10.1016/s0168-8278(97)80421-7

[23] Johnson GK Saeian K Geenen JE. Primary sclerosing cholangitis treated by endoscopic biliary dilation: review and long-term follow-up evaluation. Curr Gastroenterol Rep. 2006;8:147–155. 16533478 10.1007/s11894-006-0011-y

[24] Ringe KI Bergquist A Lenzen H Kartalis N Manns MP Wacker F. Clinical features and MRI progression of small duct primary sclerosing cholangitis (PSC). Eur J Radiol. 2020;129:109101. 32505896 10.1016/j.ejrad.2020.109101

