# Supplementary material for: Surveillance MRI is associated with improved survival in patients with primary sclerosing cholangitis
Source: Hepatol Commun. 2024 May 2;8(5):e0442. doi: 10.1097/HC9.0000000000000442 (PMC11068143; doi:10.1097/HC9.0000000000000442)

Supplementary Table 1: Cause of death stratified by surveillance status

| **Cause of death** | **MRCP Surveillance (N=20) *** | **No MRCP surveillance (N=15) **** |
| --- | --- | --- |
| End-stage liver disease | 4 (20%) | 5 (33%) |
| HBCa | 7 (35%) | 2 (13%) |
| HCC | 1 (5%) | 0 (0%) |
| Other malignancy | 0 (0%) | 4 (27%) |
| Cholangitis | 1 (5%) | 0 (0%) |
| Non-liver related | 3 (15%) | 1 (7%) |

HBCa; hepatobiliary cancer, HCC; hepatocellular carcinoma, MRCP; magnetic resonance cholangiopancreatography

*Four missing from surveillance group

** Three missing from no surveillance group

Supplementary Table 2: Indications for ERCP

| **Indication** | **MRCP Surveillance (N=42) *** | **No MRCP surveillance (N=8) **** |
| --- | --- | --- |
| Cholangitis | 11 (32.4%) | 3 (42.9%) |
| Concern for malignancy | 9 (26.5%) | 1 (14.3%) |
| Hepatolithiasis | 1 (2.9%) | 0 (0%) |
| Symptoms with cholestasis | 6 (17.6%) | 1 (14.3%) |
| Asymptomatic DS | 6 (17.6%) | 0 (0%) |
| Diagnosis of PSC | 1 (2.9%) | 2 (28.6%) |

DS; dominant stricture, MRCP; magnetic resonance cholangiopancreatography, PSC; primary sclerosing cholangitis

*Eight missing from surveillance group

** One missing from no surveillance group

Supplementary Table 3: Cox proportional hazards model univariate and multivariate analysis

| **Variable** | **Univariate** | | | **Multivariate** | | |
| --- | --- | --- | --- | --- | --- | --- |
|  | **HR** | **95% CI** | **p-value** | **HR** | **95% CI** | **p-value** |
| **MRCP Surveillance** | 0.40 | 0.19-0.81 | **0.011** | 0.29 | 0.14-0.59 | **<0.001** |
| **Age** | 1.06 | 1.04-1.09 | **<0.001** | 1.06 | 1.03-1.09 | **<0.001** |
| Cirrhosis | 2.34 | 0.51-0.99 | 0.052 |  |  |  |
| **MELD** | 1.11 | 1.05-1.16 | **<0.001** | 1.08 | 1.02-1.13 | **0.002** |
| DS | 0.85 | 0.36-2.01 | 0.723 |  |  |  |
| **CCA** | 6.98 | 3.70-13.2 | **<0.001** | 5.22 | 2.23-12.2 | **<0.001** |
| IBD | 0.53 | 0.24-1.16 | 0.114 |  |  |  |

CCA; cholangiocarcinoma, IBD; inflammatory bowel disease, MELD; Model of end-stage liver disease, MRCP; magnetic resonance imaging with cholangiopancreatography

Supplementary Table 4: Competing risks multivariable regression analysis

| **Variable** |  | | |
| --- | --- | --- | --- |
|  | **Multivariate HR** | **95% CI** | **p-value** |
| **MRCP Surveillance** | 0.33 | 0.17-0.64 | **0.001** |
| **Age** | 1.07 | 1.03-1.09 | **<0.001** |
| **MELD** | 1.08 | 1.01-1.13 | **0.015** |
| **CCA** | 5.77 | 1.91-17.4 | **0.002** |

CCA; cholangiocarcinoma, IBD; MELD; Model of end-stage liver disease, MRCP; magnetic resonance imaging with cholangiopancreatography

Supplementary Table 5: Unweighted cohort stratified by surveillance MRCP interval

|  | **Yearly (N=172)** | **Longer than yearly (N=41)** | **P-value** | **Standardized mean difference** |
| --- | --- | --- | --- | --- |
| Age of PSC diagnosis, years (median, IQR) | 33 (21-49) | 44 (22-54) | 0.084 | **0.315** |
| Male, N (%) | 114 (66.3%) | 26 (63.4%) | 0.870 | 0.060 |
| Large duct, N (%) | 13 (7.6%) | 6 (14.6%) | 0.266 | **0.225** |
| Inflammatory bowel disease, N (%) | 130 (75.6%) | 32 (78%) | 0.897 | 0.058 |
| Dominant stricture, N (%) | 49 (28.5%) | 16 (39%) | 0.259 | **0.224** |
| ERCP, N (%) | 34 (19.7%) | 7 (8.9%) | 0.259 | **0.436** |
| Cirrhosis, N (%) | 65 (37.8%) | 14 (34.1%) | 0.799 | 0.076 |
| Death, N (%) | 14 (8.1%) | 6 (14.6%) | 0.325 | **0.206** |
| LT, N (%) | 26 (15.1%) | 5 (12.2%) | 0.818 | 0.085 |
| HBCa, N (%) | 9 (5.2%) | 2 (4.9%) | 1.00 | 0.016 |
| Serum ALP*, IU/L (median, IQR) | 176 (101-318) | 142 (105-200) | 0.353 | **0.174** |
| MELD score* (median, IQR) | 8.33 (7.21-10) | 9.08 (7.5-10.8) | 0.162 | 0.015 |

ALP; alkaline phosphatase, ERCP; endoscopic retrograde cholangiopancreatography, HBCa; hepatobiliary cancer, LT; liver transplant, MELD; Model of end-stage liver disease, MRCP; magnetic resonance cholangiopancreatography

*Values taken at last follow up

Supplementary Table 6: Inverse probability treatment weighted cohorts stratified by surveillance MRCP interval

|  | **Yearly** | **Longer than yearly** | **P-value** | **Standardized mean difference** |
| --- | --- | --- | --- | --- |
| Age of PSC diagnosis, years (median, IQR) | 35 (22-50) | 28 (19-51) | 0.624 | 0.069 |
| Male, % | 65.9% | 66.6% | 0.938 | 0.014 |
| Dominant stricture, % | 30.6% | 32.1% | 0.865 | 0.031 |
| Type, % | 8.8% | 8.4% | 0.930 | 0.014 |
| IBD, % | 75.7% | 72.4% | 0.713 | 0.077 |
| Cirrhosis, N (%) | 37.3% | 38.2% | 0.927 | 0.018 |
| LT, N (%) | 15% | 17.9% | 0.730 | 0.079 |
| Serum ALP (median, IQR) | 174 (97-297) | 153 (109-248) | 0.976 | 0.027 |
| MELD score (median, IQR) | 8.23 (7.11-9.98) | 9.36 (8.28-11.94) | 0.041 | 0.097 |
| ERCP* (%) | 4.64% | 20.6% | 0.393 | **0.275** |

ALP; alkaline phosphatase, ERCP; endoscopic retrograde cholangiopancreatography, LT; liver transplant, MELD; Model of End-stage Liver Disease score, MRCP; magnetic resonance cholangiopancreatography

*Not weighted

Supplementary Figure 1: Standardized mean differences pre- and post-weighting between surveillance and no surveillance groups


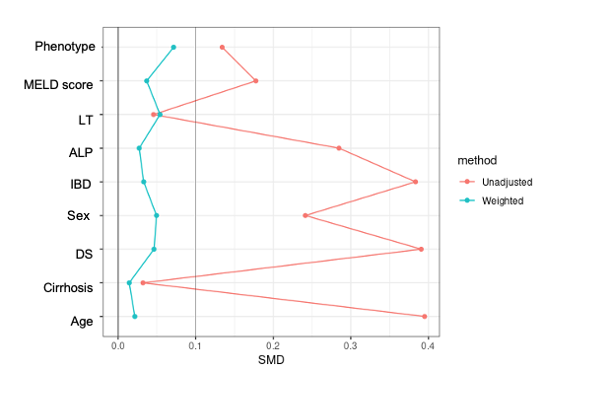


*Weighting was unable to be performed for ERCP as a covariate due to missing data for patients without a DS which would result in a much smaller population for analysis. SMD between groups for all these covariates were more than 0.10 prior to IPTW, but post-IPTW a SMD of less than 0.10 was achieved for all covariates.*

ALP; alkaline phosphatase, DS; dominant stricture, IBD; inflammatory bowel disease, LT; liver transplant, MELD; Model of end-stage liver disease, SMD; standardised mean difference

Supplementary Figure 2: Standardized mean differences pre- and post-weighting between different MRCP interval groups


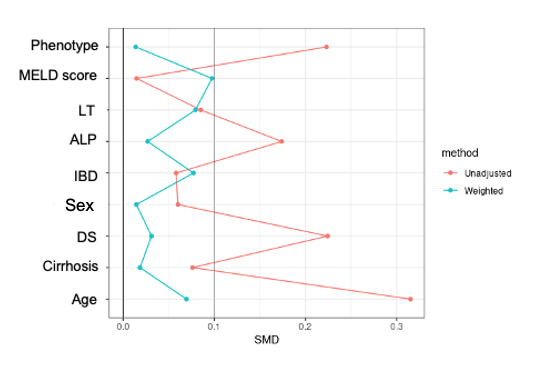


*Presence of cirrhosis was removed from IPTW matching to achieve optimal balance between groups as there was no significant difference pre- and post-weighting in both cohorts. SMD between groups for all these covariates were more than 0.10 prior to IPTW, but post-IPTW a SMD of less than 0.10 was achieved for all.*

ALP; alkaline phosphatase, DS; dominant stricture, IBD; inflammatory bowel disease, LT; liver transplant, MELD; Model of end-stage liver disease, SMD; standardised mean difference

# Further supplementary analysis

Patients with small duct PSC were excluded from the original cohort and inverse probability treatment weighting performed without phenotype at diagnosis as a confounding covariate. Overall and transplant-free survival was still improved in patients who underwent regular MRCP surveillance compared to patients who did not, with no difference in post-cancer survivorship. Results from the sub-analysis are presented in the following tables and figures.

Table 1: Demographics and clinical characteristics of unweighted cohorts

|  | **MRCP Surveillance (N=201)** | **No MRCP surveillance (N=68)** | **P-value** | **Standardized mean difference** |
| --- | --- | --- | --- | --- |
| Age at PSC diagnosis, years (median, IQR) | 36 (22-51) | 47.5 (29.8-56.5) | **0.006** | 0.391 |
| Male, N (%) | 133 (66.2) | 37 (54.4) | 0.111 | 0.242 |
| Inflammatory bowel disease, N (%) | 153 (76.1) | 40 (58.8) | **0.010** | 0.376 |
| Dominant stricture, N (%) | 64 (31.8) | 11 (16.2) | **0.020** | 0.373 |
| ERCP, N (%) | 42 (20.9) | 8 (11.7) | 0.732 | 0.258 |
| Cirrhosis, N (%) | 79 (39.3) | 28 (41.2) | 0.897 | 0.038 |
| Death, N (%) |  |  |  |  |
| LT, N (%) | 33 (16.4) | 13 (19.1) | 0.745 | 0.071 |
| HBCa, N (%) | 11 (5.5) | 2 (2.9) | 0.607 | 0.126 |
| Serum ALP*, IU/L (median, IQR) | 166 (101-292) | 112.5 (78.5-220) | **0.006** | 0.234 |
| Serum bilirubin*, μmol/L (median, IQR) | 15 (9-29) | 11 (7-18.5) | **0.005** | 0.308 |
| MELD score* (median, IQR) | 8.47 (6.62-10.9) | 7.5 (6.43-9.75) | **0.017** | 0.299 |

ALP; alkaline phosphatase, CCA; cholangiocarcinoma, ERCP; endoscopic retrograde cholangiopancreatography, GB; gallbladder adenocarcinoma, HBCa; hepatobiliary cancer, LT; liver transplant, MELD; Model of End-stage Liver Disease, MRCP; magnetic resonance cholangiopancreatography

*Values taken at last follow up

Table 2: Weighted Cox proportional hazards model univariate and multivariate analysis for overall survival

| **Variable** | **Univariate** | | | **Multivariate** | | |
| --- | --- | --- | --- | --- | --- | --- |
|  | **HR** | **95% CI** | **p-value** | **HR** | **95% CI** | **p-value** |
| **MRCP Surveillance** | 0.36 | 0.17-0.76 | **0.001** | 0.31 | 0.16-0.60 | **<0.001** |
| **Age** | 1.06 | 1.04-1.09 | **<0.001** | 1.06 | 1.03-1.10 | **<0.001** |
| **Cirrhosis** | 3.04 | 1.18-7.86 | **0.022** | 2.77 | 1.03-7.40 | **0.042** |
| **MELD** | 1.10 | 1.05-1.15 | **<0.001** | 1.08 | 0.97-1.09 | 0.254 |
| DS | 1.00 | 0.42-2.38 | 0.999 |  |  |  |
| **CCA** | 7.65 | 3.80-15.4 | **<0.001** | 5.22 | 4.52-21.5 | **<0.001** |
| IBD | 0.54 | 0.22-1.26 | 0.156 |  |  |  |

CCA; cholangiocarcinoma, IBD; inflammatory bowel disease, MELD; Model of end-stage liver disease, MRCP; magnetic resonance imaging with cholangiopancreatography

Figure 1: Weighted Kaplan-Meier curve for overall survival


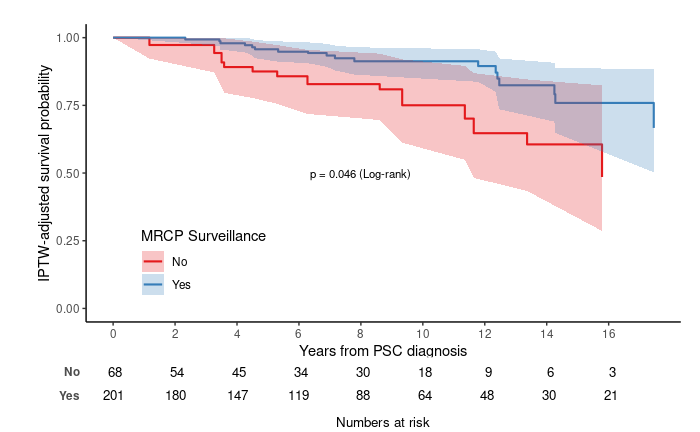


Figure 2: Weighted Kaplan-Meier curve for transplant-free survival


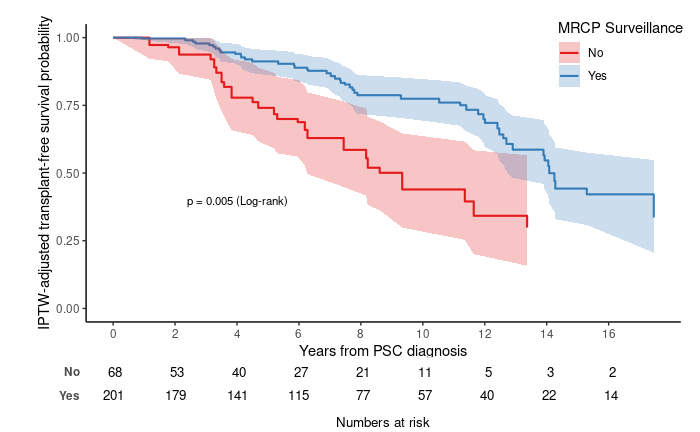


Figure 3: Weighted Kaplan-Meier curve for survival post cancer diagnosis


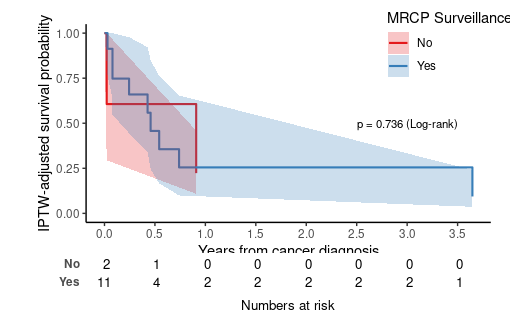

Supplement: SUPPLEMENTARY MATERIAL [file hc9-8-e0442-s001.docx]
